# Identification of genes coding for B cell antigens of *Mycoplasma mycoides *subsp. *mycoides *Small Colony (*Mmm*SC) by using phage display

**DOI:** 10.1186/1471-2180-9-215

**Published:** 2009-10-09

**Authors:** Dubravka R Miltiadou, Arshad Mather, Edy M Vilei, Dion H Du Plessis

**Affiliations:** 1 Immunology Section, Onderstepoort Veterinary Institute, Private Bag X5, Onderstepoort 0110, Republic of South Africa; 2 Institute of Veterinary Bacteriology, University of Bern, Vetsuisse Faculty, Länggass-Str. 122, PO Box, 3001 Bern, Switzerland

## Abstract

**Background:**

Contagious bovine pleuropneumonia (CBPP) is a mycoplasmal disease caused by *Mycoplasma mycoides *subsp. *mycoides *SC (*Mmm*SC). Since the disease is a serious problem that can affect cattle production in parts of Africa, there is a need for an effective and economical vaccine. Identifying which of the causative agent's proteins trigger potentially protective immune responses is an important step towards developing a subunit vaccine. Accordingly, the purpose of this study was to determine whether phage display combined with bioinformatics could be used to narrow the search for genes that code for potentially immunogenic proteins of *Mmm*SC. Since the production of IgG2 and IgA are associated with a Th_1 _cellular immune response which is implicated in protection against CBPP, antigens which elicit these immunoglobulin subclasses may be useful in developing a subunit vaccine.

**Results:**

A filamentous phage library displaying a repertoire of peptides expressed by fragments of the genome of *MmmSC *was constructed. It was subjected to selection using antibodies from naturally- and experimentally-infected cattle. Mycoplasmal genes were identified by matching the nucleotide sequences of DNA from immunoselected phage particles with the mycoplasmal genome. This allowed a catalogue of genes coding for the proteins that elicited an immune response to be compiled. Using this method together with computer algorithms designed to score parameters that influence surface accessibility and hence potential antigenicity, five genes (*abc, gapN, glpO, lppB *and *ptsG*) were chosen to be expressed in *Escherichia coli*. After appropriate site-directed mutagenesis, polypeptides representing portions of each of these proteins were tested for immunoreactivity. Of these five, polypeptides representing expression products of *abc *and *lppB *were recognised on immunoblots by sera obtained from cattle during a natural outbreak of the disease.

**Conclusion:**

Since phage display physically couples phenotype with genotype, it was used to compile a list of sequences that code for *Mmm*SC proteins bearing epitopes which were recognised by antibodies in the serum of infected animals. Together with the appropriate bioinformatic analyses, this approach provided several potentially useful vaccine or diagnostic leads. The phage display step empirically identified sequences by their interaction with antibodies which accordingly reduced the number of ORFs that had to be expressed for testing. This is a particular advantage when working with *Mmm*SC since the mycoplasmal codon for tryptophan needs to be mutated to prevent it from being translated as a stop in *E. coli*.

## Background

Contagious bovine pleuropneumonia (CBPP), a pulmonary disease caused by *Mycoplasma mycoides *subsp. *mycoides *SC (*Mmm*SC) is a major constraint to cattle production in Africa [1]. The current vaccines are not always fully effective [2] and there remains an urgent need to control or even eradicate the disease. Although the nucleotide sequence of the *Mmm*SC type strain PG1 genome is available, the proteins responsible for protection have not been identified. Accordingly, an important step towards a subunit vaccine would be to identify which of the potentially large number of antigens encoded in its genome [3-5] actually trigger immune responses during infection. Serum antibodies are likely to be involved in immunity since passive transfer of sera from recovered cattle can protect recipient calves [6,7], but Th_1 _memory lymphocytes and γδ T-cells are also active [8-10]. Identifying which antigens evoke one or more of these immune pathways therefore remains a key step in developing a subunit-based CBPP vaccine [11].

Phage display [12] makes it possible to identify antigenic proteins by using antibodies from an immune source to select binding peptides from a large repertoire of random amino acid sequences [13]. Fragmented-genome or "shotgun" display libraries [14] can directly identify genes that code for the proteins of which the immunoselected peptides form a part. Provided that artefactual binders expressed by frame-shifted or incorrectly orientated inserts are excluded [15], matching the sequence coding for an antigenic peptide with the sequence of the genome from which it is derived locates the encoding gene. The first *Mmm*SC display library was constructed by Persson and co-workers [16] and more recently, the approach was also applied to *Mycoplasma hyopneumoniae *[17] as a way of identifying immunogenic polypeptides.

To locate genes coding for potentially immunogenic proteins, enzymatically-generated fragments of *Mmm*SC chromosomal DNA were used to construct a genome-specific filamentous phage display library which was subjected to selection using antibodies from a CBPP outbreak in Botswana [18] and an experimentally infected animal from Mali designated C11 [19]. CD4^+ ^T-cell activation and IFNγ release are associated with an IgG2 humoral immune response [20] while IgA is associated with local mucosal immunity. Accordingly, both immunoglobulin classes were used separately to select peptides as well as using total IgG. Using this approach together with computer algorithms designed to identify linear B-cell epitopes [21], five genes were chosen to be expressed for further analysis and testing to establish whether they were recognised by sera from cattle obtained during a natural outbreak of the disease.

## Results

### Construction of a fragmented-genome library

A pIII fusion protein phage display library of approximately 4 × 10^5 ^primary clones displaying peptides derived from the *Mmm*SC genome was constructed by ligating DNA fragments ranging in size from approximately 30 to 900 bp as determined by agarose gel electrophoresis into a filamentous phage display vector. The probability of the genome being represented was 0.97 if the average insert size was 240 bp. DNA sequencing of 16 arbitrarily-chosen clones showed no obvious bias towards any particular region of the mycoplasmal genome. Of the 16, two copies of one of the sequenced DNA inserts were in-frame and in the correct orientation. The largest insert was 178 base pairs and the smallest 52.

**Figure 1:**
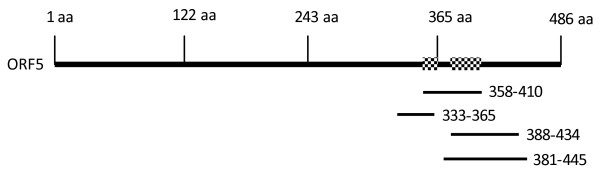
**Schematic representation showing alignment of the hypothetical proline-rich glycoprotein ORF5 with selected phage fusion peptides**. Antigenic regions suggested by the presence of overlapping sequences located between amino acid residues 358-365 and 388-410 are indicated by shading.

To verify that the library was large and diverse enough to identify other unknown *Mmm*SC antigens, it was first screened in a defined model system by panning on immuno-purified IgG prepared from a rabbit immune serum directed against amino acid residues 328-478 of the proline-rich *Mmm*SC glycoprotein which is coded for by ORF5 (EMBL/GenBank accession number CAE77151). Multiple copies of overlapping peptides that mapped to a defined region on the target glycoprotein spanning residues 333 to 445 were selected (Figure 1).

### Identification of antigenic peptides

**Figure 2:**
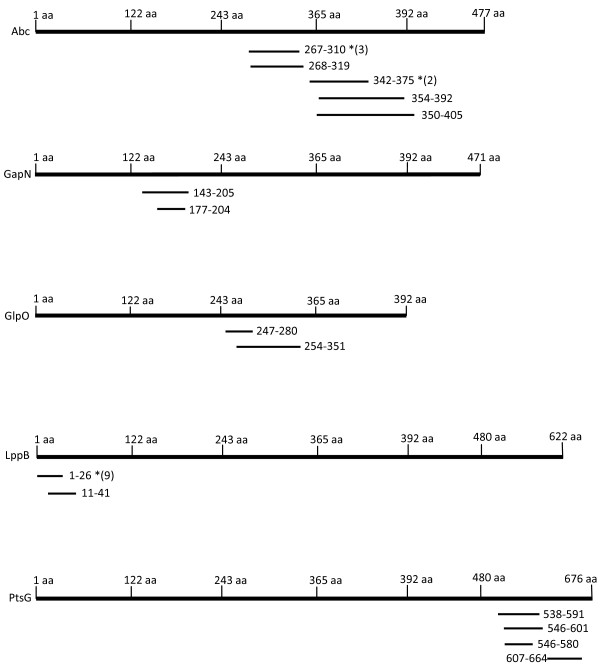
**Schematic representation showing alignment of the five selected proteins with phage displayed peptides obtained with CBPP immune sera**. The entire open reading frames that code for the five proteins (Abc, GapN, GlpO, LppB and PtsG) selected for expression aligned with the phage displayed immunogenic polypeptides that were isolated by panning the fragmented *Mmm*SC genome phage display library against CBPP immune sera. Asterisks indicate peptides isolated more than once and the numbers in parentheses specify the number of repeated isolations for such peptides.

Panning experiments aimed at selecting phage displayed peptides that were recognised by the total serum IgG, the IgG2 component and the IgA subpopulation of animal C11 and pooled serum from seven cattle affected by a CBPP outbreak in Botswana were performed. Clones selected by each immunoglobulin class were randomly chosen for DNA sequencing. After panning, all clones were in-frame and without stops. Fifteen peptides recognised by the total IgG preparation from the serum of animal C11 matched regions in eight different *Mmm*SC proteins in the EMBL/GenBank database, while 29 peptides that had been selected using IgG2 corresponded with regions on 17 different proteins (Table 1). The library was not panned with total serum IgG from Botswana but peptides were selected using IgG2 which matched parts of four of the proteins identified by the C11 antiserum (GyrB, GlpO, LppB and Abc). Bronchoalveolar lavage (BAL) IgA from animal C11 specifically selected four peptides that could be correlated with portions of *Mmm*SC proteins while the Botswana serum selected six peptides that matched three *Mmm*SC proteins. A single C11 IgG2 peptide matched *Mmm*SC glyceraldehyde-3-phosphate dehydrogenase (GapN) which was also identified by one C11 IgA peptide. Four peptides that were isolated with C11 IgG2 matched the ABC transporter, substrate-binding component protein (Abc). This protein was also identified by the IgG from Mali (i.e. animal C11) and IgG2 and IgA from the Botswana serum pool. Glycerol-3-phosphate oxidase (GlpO) was selected by peptides from both C11 and Botswana serum IgG2. Three of the Botswana IgA peptides matched to the mycoplasmal prolipoprotein B (LppB) amino acid sequence. This protein was also selected with both the C11 and Botswana IgG2 serum antibodies. The PTS system, glucose-specific IIBC component (PtsG) was identified by four peptides (Figure 2).

**Table 1:** List of phage displayed peptides and their corresponding *Mmm*SC proteins identified after selection with the indicated antibody subpopulations from animal C11 and the Botswana outbreak

*Mmm *SC protein match	Identified by serum from:		Accession number^a^	Locus Tag
			
	Animal C11^c^	Botswana outbreak^c^		
**IgG panning**				
Putative variable surface protein	Yes (3)	ND	CAE77433	MSC_0818
Cell division protein (FtsZ)	Yes (1)	ND	CAE77211	MSC_0588
ABC transporter, substrate-binding component (Abc)	Yes (1)	ND	CAE77420^b^	MSC_0804
Prolipoprotein (Lpp)	Yes (1)	ND	CAE77256	MSC_0635
D-lactate dehydrogenase (Ldh)	Yes (2)	ND	CAE76687	MSC_0034
DNA gyrase subunit A (GyrA)	Yes (2)	ND	CAE76660	MSC_0007
DNA gyrase subunit B (GyrB)	Yes (1)	ND	CAE76659	MSC_0006
PTS system, glucose-specific, IIBC component (PtsG)	Yes (4)	ND	CAE77485^b^	MSC_0873
				
**IgG2 panning**				
Transposase IS*Mmy1*C	Yes (1)	No	CAE77424	MSC_0808
30S ribosomal protein S5 (RpsE)	Yes (1)	No	CAE77346	MSC_0728
Putative glucose-specific IIABC component (PtsG)	Yes (1)	No	CAE76806	MSC_0161
Prolipoprotein (Lpp)	Yes (1)	No	CAE77256	MSC_0635
Prolipoprotein LppC (LppC)	Yes (1)	No	CAE76772	MSC_0122
Alanine-tRNA ligase (AlaS)	Yes (1)	No	CAE76823	MSC_0178
DNA gyrase subunit B (GyrB)	Yes (1)	Yes (1)	CAE76659	MSC_0006
Methionine adenosyltransferase (MetK)	Yes (1)	No	CAE77120	MSC_0492
Glyceraldehyde-3-phosphate dehydrogenase (GapN)	Yes (1)	No	CAE77137^b^	MSC_0509
Glycerol-3-phosphate oxidase (GlpO)	Yes (1)	Yes (1)	CAE76900^b^	MSC_0259
D-lactate dehydrogenase (Ldh)	Yes (1)	No	CAE76687	MSC_0034
P115-like protein with SMC_C motif	Yes (7)	No	CAE77104	MSC_0476
Ribose/Galactose ABC transporter, ATP-binding component (RbsA)	Yes (3)	No	CAE76663	MSC_0010
Prolipoprotein B (LppB)	Yes (2)	Yes (5)	CAE77147^b^	MSC_0519
Glucose inhibited division protein A (GidA)	Yes (1)	No	CAE77646	MSC_1042
ABC transporter, substrate-binding component (Abc)	Yes (4)	Yes (1)	CAE77420^b^	MSC_0804
Fructose-bisphosphate aldolase class II (FbaA2)	Yes (1)	No	CAE76786	MSC_0139
				
**IgA panning**				
Glyceraldehyde-3-phosphate dehydrogenase (NADP) (GapN)	Yes (1)	No	CAE77137^b^	MSC_0509
Prolipoprotein B (LppB)	No	Yes (3)	CAE77147^b^	MSC_0519
ABC transporter, substrate-binding component (Abc)	No	Yes (2)	CAE77420^b^	MSC_0804
CTP synthase (CtrA)	Yes (1)	No	CAE76781	MSC_0134
Prolipoprotein LppC (LppC)	No	Yes (1)	CAE76772	MSC_0122
Thioredoxin reductase (NADPH) (TrxB)	Yes (1)	No	CAE77547	MSC_0938
Asparagine-tRNA ligase (AsnS)	Yes (1)	No	CAE76732	MSC_0080

^a ^EMBL/GenBank accession numbers for the protein sequences.

^b ^Proteins that were expressed in this study (see Figure 3).

^c ^Figures in parenthesis indicate numbers of peptides selected

ND - not determined

### Expression and antigenicity of MmmSC polypeptides

Given the difficulty of easily expressing recombinant mycoplasmal proteins due to codon usage, a short list of genes to be expressed in *E. coli *was compiled. Based on predetermined selection criteria discussed below and using algorithms for antigenicity and surface location together with knowledge of their established characteristics, this list comprised the ABC transporter Abc, GapN, GlpO, LppB and PtsG. Site-directed mutations allowed 62% of the ABC transporter Abc (residues 184 to 477), 96% of GlpO (residues 6 to 380) and 61% of LppB (residues 1 to 376) to be expressed. Fifty percent of GapN (residues 88 to 322) and 38% of the PtsG (residues 421 to 676) polypeptides could be expressed without mutating (Figure 2).

**Figure 3:**
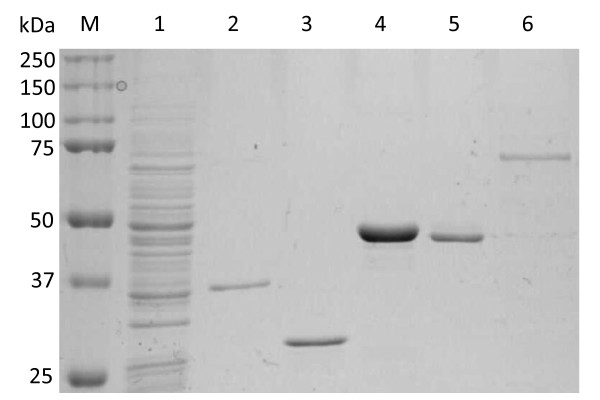
**Expression of the five selected proteins in *E. coli***. SDS-PAGE (10%) showing segments of the protein antigens that were expressed in *E. coli*. Lanes: M, molecular mass standards; 1, 12 μg of total antigen of *Mmm*SC strain 8740; 2-6, expressed segments of proteins Abc, GapN, GlpO, LppB and PtsG, respectively.

Four of the resulting recombinant polypeptides were found in the *E. coli *insoluble pellet fraction. Their *M*_*r*_s were within the range expected from their amino acid sequences. The calculated *M*_*r*_s were: ABC transporter Abc, *M*_*r *_~33 kDa; GapN, *M*_*r *_~26 kDa; GlpO, *M*_*r *_~41 kDa; and LppB, *M*_*r *_43 ~kDa (compare with Figure 3). PtsG was isolated from the soluble fraction using nickel chelation, but it manifested in PAGE as two bands with *M*_*r*_s ~70 and ~45 kDa (Figure 3; calculated *M*_*r *_~28 kDa).

**Figure 4:**
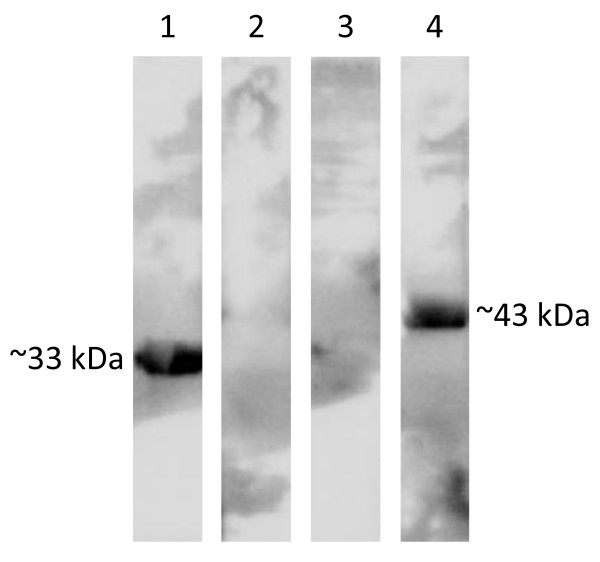
**Chemiluminescent immunoblot**. Recognition of the ABC transporter Abc (lane 1) and lipoprotein LppB (lane 4) polypeptides that were expressed in *E. coli *by a pool of sera obtained from cattle that were naturally infected with CBPP during the 1995 Botswana outbreak. The GapN (lane 2) and GlpO (lane 3) polypeptides were not recognised in this test format.

The pool of the seven sera obtained from the Botswana outbreak was also used in immunoblotting. The pool reacted with the expressed Abc and LppB polypeptides (Figure 4). The PtsG polypeptide bands were probed separately with serum obtained from an experimental infection. This immunoblot, however, showed multiple bands that apparently reacted with the pooled sera (not shown).

## Discussion

When a pathogen infects an animal, its epitopes leave an "imprint" in the form of a spectrum of disease-specific antibody paratopes in the serum. Most animals are therefore likely to have antibodies directed against a large number of foreign epitopes. The strategy pursued in this study was to use this complex mixture of antibodies to select binders from a limited repertoire of sequences derived from the genome of *MmmSC*, thereby focussing the phage display selection process on relevant epitopes. These binders were matched to open reading frames present in the genome. Unlike immunoblotting, this approach also identified the genes that coded for the antigenic proteins.

The fragmented genome library covered approximately 97% of the mycoplasmal genome. While adequate for its purpose, it cannot, however, be considered to have been completely random since among the 1016 proteins encoded in the genome of *Mmm*SC type strain PG1, 797 (78.4%) contain at least one UGA_trp _codon, which is read as stop codon in *E. coli*. Moreover, the frequency of UGA_trp _codons in coding sequences of *Mmm*SC genes is relatively high: 1.00% in contrast to 0.05% of UGG_trp _codons. This means that epitopes containing such stops could be disrupted. Moreover, in a phage display system, the secreted phages would be unlikely to display large oligopeptides or those that resisted being transported through the bacterial membrane or periplasm. The functionality of the library was therefore first tested by selecting peptides with a polyclonal rabbit antiserum directed against a single recombinant *Mmm*SC polypeptide, i.e., the C-terminal proline-rich portion of ORF5. The ORF5 gene product [22] corresponds to the 486 aa protein having EMBL/GenBank accession number CAE77151 [5]. The ORF5 antiserum selected a series of overlapping peptides thereby identifying a B cell epitope and confirming that polyclonal serum could specifically select antigenic peptides from the phage displayed repertoire. A further important indication that the peptides had been specifically selected was that prior to panning, only 12.5% of the sequenced inserts contained in the library were both in-frame and in the correct orientation for translation as mycoplasmal peptides. In contrast, after panning, all were in-frame and without stops. This finding, together with the way in which immunoselection yielded multiple copies of some peptides (particularly those that overlapped but were not identical), provided additional evidence that the strategy was essentially sound.

While 26 different *Mmm*SC genes matched sequences selected by phage display, those chosen for expression in *E. coli *were required to have fulfilled criteria which were considered to have a bearing on their usefulness as possible vaccine antigens. Firstly, since the pathogen enters the animal via the nasal passages, preference was given to genes selected by IgA from Mali and Botswana. Secondly, only genes that were identified by multiple overlapping copies of each phage displayed peptide qualified. Thirdly, peptides that fulfilled the first two criteria, but which were selected with a negative bovine serum were excluded. Finally, the protein's likely function or structural position was taken into account with a focus on previously-identified membrane-associated proteins [23] which also fulfilled antigenicity criteria as predicted by bioinformatics analyses. Although not excluded as being potentially useful, any overlapping sequences that coded for internally located proteins e.g. the DNA gyrase subunit B (Table 1) were not investigated in this study. Applying these criteria allowed us to focus on the ABC transporter, substrate-binding component protein (Abc), the glyceraldehyde-3-phosphate dehydrogenase (GapN), the glycerol-3-phosphate oxidase (GlpO), the prolipoprotein B (LppB) and the PTS system, glucose-specific IIBC component (PtsG) for expression ***i****n E. coli*. By applying these criteria we do not exclude further studies on any of the other apparently antigenic proteins as vaccine or diagnostic targets. Even though the proliporotein LppC fulfilled our criteria, some of the peptides which matched the amino acid sequence included sequences of unknown origins which did not align with the target ORF (not shown).

ABC transporter proteins act on a wide variety of substrates that include sugars, peptides, proteins and toxins [24]. For example, the ATP-binding cassette (ABC) transporter GtsABC together with GlpO forms part of the glycerol catabolism pathway associated with *Mmm*SC virulence [25,26]. GlpO itself has been identified as a significant *Mmm*SC virulence factor [27-29]. GapN, in contrast, may play a role in transcription [30] and apoptosis [31]. Membrane lipoproteins that interact with host cells can stimulate the release of pro-inflammatory cytokines [32] and are major antigens [23,33,34]. The lipoprotein (LppB) identified by the phage display is found in African and Australian strains of *Mmm*SC, but not in the less virulent European strains [22]. The *ptsG *gene, which occurs in duplicate in many *Mmm*SC strains [35], encodes the permease of the phosphoenolpyruvate:glucose phosphotransferase system. It has also been implicated in intraclonal antigenic variation [36], a possible factor in the evasion of the host immune response. With the exception of GapN, these proteins are likely to be involved in pathogenicity or to be accessible to B cell receptors. They therefore have potential either in vaccine or diagnostics development. Only two of the expressed polypeptides, however, reacted in immunoblots, possibly because their epitopes in the denatured state most faithfully resembled the phage displayed peptides that were originally bound in the selection process. Although phage display of necessity identified B cell epitopes, it is not yet clear whether it is this response, or a cell-mediated one based on CD4 [37], which is a primarily responsible for protection. The proteins identified using phage display will therefore also need to be tested for their ability to cause primed lymphocytes to proliferate and produce IFNγ.

## Conclusion

Constructing a phage library that displays peptides derived from the actual organism of interest made it possible to narrow the search for genes that code for antigenic and hence potentially immunogenic proteins of the mycoplasma that causes CBPP. Because of their interaction with antibodies in the serum of infected animals, these proteins may be regarded as potential vaccine targets, in particular those selected using IgG2 and IgA. A model epitope discovery system has shown that many antigenic peptides obtained from such phage libraries have potential as vaccine antigens [38]. It may therefore also be worth examining the actual antigenic *Mmm*SC peptides that were selected from the epitope library as possible components of a subunit vaccine. Knowing which proteins are antigenic may help to identify targets for generating knockout mutants for use as genetically defined vaccines [39]. Lastly, phage display was able to identify polypeptides that were recognised in immunoblotting by serum from animals that were affected by a natural disease outbreak. As well as having potential as vaccine antigens, such peptides may be useful diagnostic targets.

## Methods

### Strains, growth conditions and vectors

*MmmSC *strain 8740 from Cameroon, provided by Dr. L. Dedieu, CIRAD-EMVT, Montpellier, France, was cultured in PPLO broth medium (Difco, Detroit, MI, USA) containing thallium acetate (1% w/v), ampicillin (0.5 mg/ml), heat-inactivated horse serum (20%) [40] and supplemented with 0.1 volumes of 25% fresh yeast extract. Mycoplasmas were grown at 37°C in 5% CO_2 _until stationary growth phase and harvested by centrifugation at 20000 *g *for 20 min. For genetic manipulation and subcloning, *E. coli *strains TG1 (Stratagene, La Jolla, CA, USA), DH5α, Top10 (Invitrogen, Carlsbad, CA, USA) and BL21 Star™ (DE3) (Invitrogen) were used. The phage display vector fdtet 8.53 was a gift from Dr. V. K. Chaudhary, University of Delhi, New Delhi, India.

### Antisera, antibodies, and immunoblot analysis

Anti-ORF5 immune serum was obtained by injecting rabbits with amino acid residues 328-478 of the 486 aa proline-rich *Mmm*SC ORF5 [22]. Bovine sera and bronchoalveolar lavage (BAL) from animals C11 (recovered from a sub-acute to chronic experimental infection) and T1 (uninfected control) were from Dr. M. Niang, Central Veterinary Laboratory, Bamako, Mali [4,19]. The seven bovine sera used in screening and immunoblotting were a kind gift from the Botswana National Veterinary Laboratory in Gabarone, Botswana [18]. Antibodies were isolated using ImmunoPure^® ^Protein G columns (Pierce, Rockford, IL, USA). Antibody-containing fractions were applied to Excellulose™ GF-5 Desalting columns (Pierce). Before selection by panning, unwanted filamentous phage antibodies were removed from the C11 serum by cross-absorption [41]. BAL IgA from animal C11 and serum IgA from Botswana cattle were used in pannings, but a limited volume was available and the samples were not cross-absorbed. Negative control pannings using BAL IgA and total IgG from the control animal (T1) were also performed.

Immunoblotting was performed according to standard protocols. A volume of 10 μl of each of the seven sera from Botswana were added to 5 ml of 1% milk powder (MP) suspended in PBS, pH 7.4. Blots were incubated overnight in the pool of diluted sera at room temperature. For the detection of bound antibodies, sheep horseradish peroxidise conjugated anti-bovine IgG (catalogue No. PP200; The Binding Site, Birmingham, UK) was diluted 1:10000 and incubated with the blot for an hour at room temperature. Bound antibodies were detected after incubation of the blot with SuperSignal^® ^West Pico chemiluminescent substrate (Pierce) using the Lumi-Imager from Roche Molecular Biochemicals.

### Display library construction

Phage library construction using the pIII phage display vector fdtet 8.53 was as described by Gupta and co-workers [42]. This entailed ligating blunt-ended fragments of *Mmm*SC genomic DNA in the presence of the restriction enzyme *Srf*I and T4 DNA ligase. The extent to which the genome was represented in the primary library with a theoretical probability of 0.99 was calculated using the method of Clarke and Carbon [43]. To deplete the resulting phage repertoire of any peptides that may have been susceptible to binding by irrelevant antibodies present in healthy bovine serum, a 50 μl volume was incubated with 2 mg of naïve bovine IgG at 4°C overnight. Any phage/IgG aggregates were removed by centrifugation for 5 min at 15000 *g *and the supernatant transferred to a new tube. The depleted library was stored at 4°C.

### Affinity selection from the phage library

The peptide display library was subjected to three successive rounds of affinity selection essentially as described [15]. For selection of fusion phages from the library with IgG2a or IgA antibodies, the polystyrene Petri dish (Falcon 1007; Becton Dickinson, Lincoln Park, NJ, USA) used for panning was first coated with antibodies specific for the desired bovine immunoglobulin subclass at a concentration of approximately 20 μg/ml before the blocking step.

### Identification of antigens

Sequences of phage displayed peptides were compared with the EMBL/GenBank database using the BLAST programs [44]. Flexibility, hydrophilicity, polarity and surface properties were scored using the programs Bcepred http://www.imtech.res.in/raghava/bcepred/ and BepiPred http://www.cbs.dtu.dk/services/BepiPred/[21,45].

### Cloning, site-directed mutagenesis, expression and purification of proteins

For expression, the relevant sequences of the targeted genes were amplified from genomic DNA and cloned in the pET100/D-TOPO^® ^*E. coli *expression vector (Invitrogen), or in the case of PtsG, in the pQE-TriSystem His·*Strep *2 vector (Qiagen). Site-directed mutagenesis (QuikChange Site-Directed mutagenesis kit; Stratagene) was used to change mycoplasmal UGA_trp _codons to *E. coli *UGG_trp _codons. Transformed *E. coli *cells were inoculated into Overnight Express Instant TB medium from Novagen (Madison, WI, USA). Following overnight induction, bacterial cells were lysed using Novagen BugBuster^® ^reagent, after which the supernatant fluids and cell pellets were analysed by SDS-PAGE and immunoblotting on a PVDF membrane using standard protocols. Proteins for PAGE analysis were purified by using ProBond nickel chelate chromatography kits as described by the manufacturer (Invitrogen).

## Authors' contributions

The first two authors made equivalent contributions to the study; DRM constructed and screened the epitope library. She was also responsible for mutating and expressing four of the genes as well as testing the resulting polypeptides in immunoblotting. AM identified, mutated and expressed the *ptsG *gene in *E. coli *and analysed and correlated the data after DRM left the Onderstepoort Veterinary Institute. EMV identified and expressed ORF5 and raised the rabbit immune serum. DHD conceptualised the study, supervised all facets of the research and is responsible for the manuscript as submitted. The authors have read and approved the final version.
